# Home caregiver strategies for feeding older adults with dysphagia after dehospitalization*

**DOI:** 10.1590/1980-220X-REEUSP-2023-0318en

**Published:** 2024-05-24

**Authors:** Mariana Souza Belmonte, Larissa Chaves Pedreira, Nildete Pereira Gomes, Daniele Vieira Oliveira, Ana Carla Filgueira de Souza e Souza, Ivana Santos Pinto

**Affiliations:** 1 Universidade Federal da Bahia, Programa de Pós-Graduação em Enfermagem e Saúde. Salvador, BA, Brazil.; 2 Universidade Federal da Bahia, Hospital Universitário Professor Edgard Santos. Salvador, BA, Brazil.

**Keywords:** Caregivers, Aged, Deglutition Disorders, Transitional Care, Cuidadores, Anciano, Trastornos de Deglución, Cuidado de Transición, Cuidadores, Idoso, Transtornos de Deglutição, Cuidado Transicional

## Abstract

**Objective:**

To understand caregivers’ strategies for offering food to older adults with oropharyngeal dysphagia after dehospitalization.

**Method:**

Qualitative research carried out with caregivers of older adults with oropharyngeal dysphagia, who were discharged after hospitalization at a university hospital in Bahia. Data collection was carried out between January and February 2023 through a semi-structured interview, whose data were organized based on content analysis and analyzed with the help of IRaMuTeQ software.

**Results:**

Three categories emerged: Caregivers’ strategies for safely offering food to older adults with dysphagia; Caregiver strategies for oral hygiene for older adults; Recognition of continuity of speech therapy after dehospitalization.

**Conclusion:**

Caregivers’ strategies for offering food to older adults with oropharyngeal dysphagia were supported by tacit knowledge and effective care in the hospital-home transition.

## RESUMO

**Objetivo:**

Apreender as estratégias de cuidadores para oferta do alimento à pessoa idosa com disfagia orofaríngea após desospitalização.

**Método:**

Pesquisa qualitativa, realizada com cuidadores de pessoas idosas com disfagia orofaríngea, que tiveram alta após internamento em um hospital universitário na Bahia. A coleta de dados foi realizada entre janeiro e fevereiro de 2023, por meio de uma entrevista semiestruturada, cujo dados foram organizados com base na análise de conteúdo e analisados com auxílio do *software* IRaMuTeQ.

**Resultados:**

Emergiram três categorias: Estratégias dos cuidadores para oferta segura do alimento à pessoa idosa com disfagia; Estratégias de cuidadores para higiene oral da pessoa idosa; Reconhecimento da continuidade do acompanhamento fonoaudiológico após desospitalização.

**Conclusão:**

As estratégias dos cuidadores para a oferta do alimento a pessoas idosas com disfagia orofaríngea foram sustentadas pelo conhecimento tácito e por um cuidado efetivo na transição hospital-domicílio.

## RESUMEN

**Objetivo:**

Comprender las estrategias de los cuidadores para ofrecer alimentos a ancianos con disfagia orofaríngea después de la deshospitalización.

**Método:**

Investigación cualitativa, realizada con cuidadores de ancianos con disfagia orofaríngea, que fueron dados de alta después de su internación en un hospital universitario de Bahía. La recolección de datos se realizó entre enero y febrero de 2023, a través de una entrevista semiestructurada, cuyos datos fueron organizados a partir del análisis de contenido y analizados con ayuda del software IRaMuTeQ.

**Resultados:**

Surgieron tres categorías: Estrategias de los cuidadores para ofrecer alimentos de forma segura a ancianos con disfagia; Estrategias del cuidador para la higiene bucal de personas mayores; Reconocimiento de la continuidad de la logopedia después de la deshospitalización.

**Conclusión:**

las estrategias de los cuidadores para ofrecer alimentos a ancianos con disfagia orofaríngea fueron sustentadas en conocimientos tácitos y cuidados efectivos en la transición hospital-hogar.

## INTRODUCTION

Caring for someone with dysphagia can be an intensive task that requires constant time and attention. This can impact caregivers’ professional and social life, leading to possible changes in their daily routines^(1)^. Thus, oropharyngeal dysphagia (OPD) is a disorder that affects the efficiency and safety of the swallowing process, due to the inability to form and transfer the food bolus safely from the mouth to the esophagus. In the hospitalization process, OPD for older adults is a complex and peculiar event^(2)^ that can lead to malnutrition and lung impairment, increasing the risk of mortality, in addition to causing anxiety and depression, affecting their quality of life, general well-being, removing the subject from their social and family environment and affecting their dependence and autonomy^(1,2,3,4)^.

Therefore, it is essential to have a healthcare team that recognizes the importance of involving caregivers from the beginning of the hospitalization process until the moment of hospital discharge, understanding what it is like to deal with the specific nutritional needs of older adults with dysphagia. National and international studies indicate that the challenges of preparing different meals, limiting shared meals as well as emotional fatigue in the face of the situation can affect caregivers’ health and well-being and the quality of care offered^(1,5,6,7,8,9)^. Considering the above, the study is justified by the need to transition from the hospital environment to the home environment, which is, in fact, a critical and challenging moment, especially for older adults and their caregivers. This process involves the need to ensure that an older adult receives the necessary care continuously and safely after discharge from hospital. To minimize the impacts of this transition and ensure timely resolution of problems, it is essential to adopt adequate hospital discharge planning involving caregivers in this care process^(10)^.

In this context, healthcare professionals need to carry out a comprehensive health status assessment of older adults with OPD before discharge, considering clinical conditions, nutritional needs, functional capacity and self-care skills as well as involving caregivers in the development of adaptations to older adults’ specific needs, with the aim of ensuring an effective and safe approach to nutrition and general care^(1,11)^. Therefore, strategies such as information sharing, printed care plans and educational resources can assist in this process, in addition to supporting care at different levels of care, especially after hospitalization^(10)^.

The present study gives visibility to the object “offering of food by caregivers to people with OPD”, given the lack of research investigating the topic, in the context of continuity of care between the hospital and the home. In clinical practice, it is observed, especially in neurological units, caregivers of older adults who are discharged from hospital with OPD, with many insecurities about offering food, whether orally or through a device such as an enteric tube or gastrostomy. By recognizing the urgency of research that addresses the topic, this study aimed to understand caregivers’ strategies for offering food to older adults with OPD after dehospitalization.

## METHOD

### Study Design

This section has a qualitative, descriptive and exploratory approach. The study meets the COnsolidated criteria for REporting Qualitative research (COREQ) recommendations^(12)^.

### Place

The research was carried out in two settings. The first setting was the neuromusculoskeletal ward of a university hospital, according to the unit’s demand for care, which has as its profile patients with neurological conditions undergoing clinical treatment, whose main sequels is functional dependence. The second setting was older adults’ home, after discharge from the hospital, when care began to be carried out or assisted by a person, usually a family member, who took over the role of caregiver.

### Population

Eight caregivers of older adults with OPD and needs for functional support, whether family or not, who were admitted to the ward and were discharged home from hospital, participated in the study. Identification was carried out by a speech therapist at the service, who recorded the classification of dysphagia and guidance on how to offer food in the medical record.

Initially, ten participants with this profile were identified and were discharged from hospital. Everyone was invited to participate in the research, where the project was presented and explained, but two withdrew due to not having access to the internet.

### Inclusion and Exclusion Criteria

Caregivers, over the age of 18, (aged 60 years or over) admitted to the neuromusculoskeletal ward of the university hospital between January and February 2023, were included. These older adults should have OPD identified by the hospital’s speech therapy team and recorded in the medical record during the period of hospitalization.

Caregivers whose messages were sent three times (morning, afternoon and night) via messaging app, but were not responded to 24 hours after hospital discharge, were excluded. The purpose of this message was to schedule the first call to begin data collection.

### Data Collect

Data collection took place in January and February 2023, supported by a health team. At the time of admission, older adults and their caregivers were approached, when the project was presented and doubts were clarified, and those who agreed to participate signed the Informed Consent Form (ICF). The interviews with caregivers were carried out by the responsible researcher remotely, via WhatsApp^®^, and everyone was informed about the recording of dialogue. Two separate interviews were carried out: the first, 24 hours after discharge, and the second, one month after hospital discharge, ensuring that the call occurred at an opportune time. It was requested that, at this time, the caregivers were in a private place, providing privacy. A semi-structured script was prepared for the interview, consisting of two sections: the first contains questions related to sociodemographic characteristics of caregivers and older adult under care, in addition to clinical aspects of caregivers and older adults; the second contains guiding questions related to the object of the study, aiming to gather information regarding the strategies adopted in the last month to offer food. The interviews were interrupted when the data was saturated.

The interviews were subjected to a pre-test phase with two people, with the same health problems, but from another hospital. At this point, it was possible to identify what could be improved in the questionnaire and changes were made. The interviews were recorded using a digital recorder and lasted approximately 50 minutes.

### Data Analysis

Data were organized and interpreted through content analysis^(13)^ in three stages: pre-analysis, with text skimming of statements, seeking a first impression of the data; material exploration, coding stage and classification into categories, with exhaustive reading of statements, identifying the central ideas; inferential interpretations, when there was exploration and discussion of central ideas based on relevant literature.

To break down the categories, IRaMuTeQ^(14)^ (*Interface de R pour les Analyzes Multidimensionnelles de Textes et de Questionnaires*) version 0.7 alpha 2, which is free and open source, was used to better understand the content of the interviews through five available types of analysis: word cloud; similarity analysis; Descending Hierarchical Classification (DHC); Correspondence Factor Analysis (CFA); and classical lexicographic analyses. CFA is an exploratory categorical data tool used to explore the association between variables, such as words, and represent this association on a Cartesian plane.

Initially, the analysis of textual data occurred in three stages: 1) text *corpus* preparation and coding with the description of the material originating from the interviews; 2) text data processing in the IRaMuTeQ software; 3) interpretation of findings by researchers.

### Ethical Aspects

This research is part of a matrix project entitled “*Cuidado transicional hospital-domicílio a pessoas adultas e idosas*”, approved by the Research Ethics Committee of the *Universidade Federal da Bahia Hospital Universitário Professor Edgar Santos* (REC-HUPES) under Opinion 5,282,090 on March 9, 2022.

The research respected the ethical aspects that permeate Resolution 466/12, and those who agreed to participate received and signed the ICF in two copies in person. To guarantee anonymity, participants had their names replaced with the names of flowers, understanding that both convey tranquility, delicacy, love, but also fragility.

## RESULTS

Eight caregivers aged between 30 and 65 years participated in the study, with an average of 42.1 years. All declared themselves to be black, with the majority being female (n = 7), Catholic (n = 5), with a family income of one minimum wage (n = 7) and completed high school (n = 6). The length of care for older adults varied between six and 24 months, and four caregivers reported not feeling safe offering food to older adults, whether orally or using a feeding device.

Caregivers were adults who cared for older adults with OPD. Four older adults who received care, after hospitalization, no longer ate orally and began to receive food exclusively through an alternative long-term feeding route (gastrostomy). The others, after discharge, continued to eat orally, starting to eat foods with a homogeneous pasty consistency, which is common in cases of dysphagia, to avoid choking and bronchoaspiration. One of them received private speech therapy, after a caregiver’s request, and managed to evolve the food consistency into a heterogeneous pasty, with introduction of soft solids in some feeding shifts.

Furthermore, in relation to an older adult who had removed his gastrostomy alone for the second time at home, the family chose, on their own, to return to oral feeding, in a homogeneous pasty consistency, using a bottle to offer food, as they believed it was safer for older adults. This was carried out without any professional guidance or assessment.

**Figure 1:**
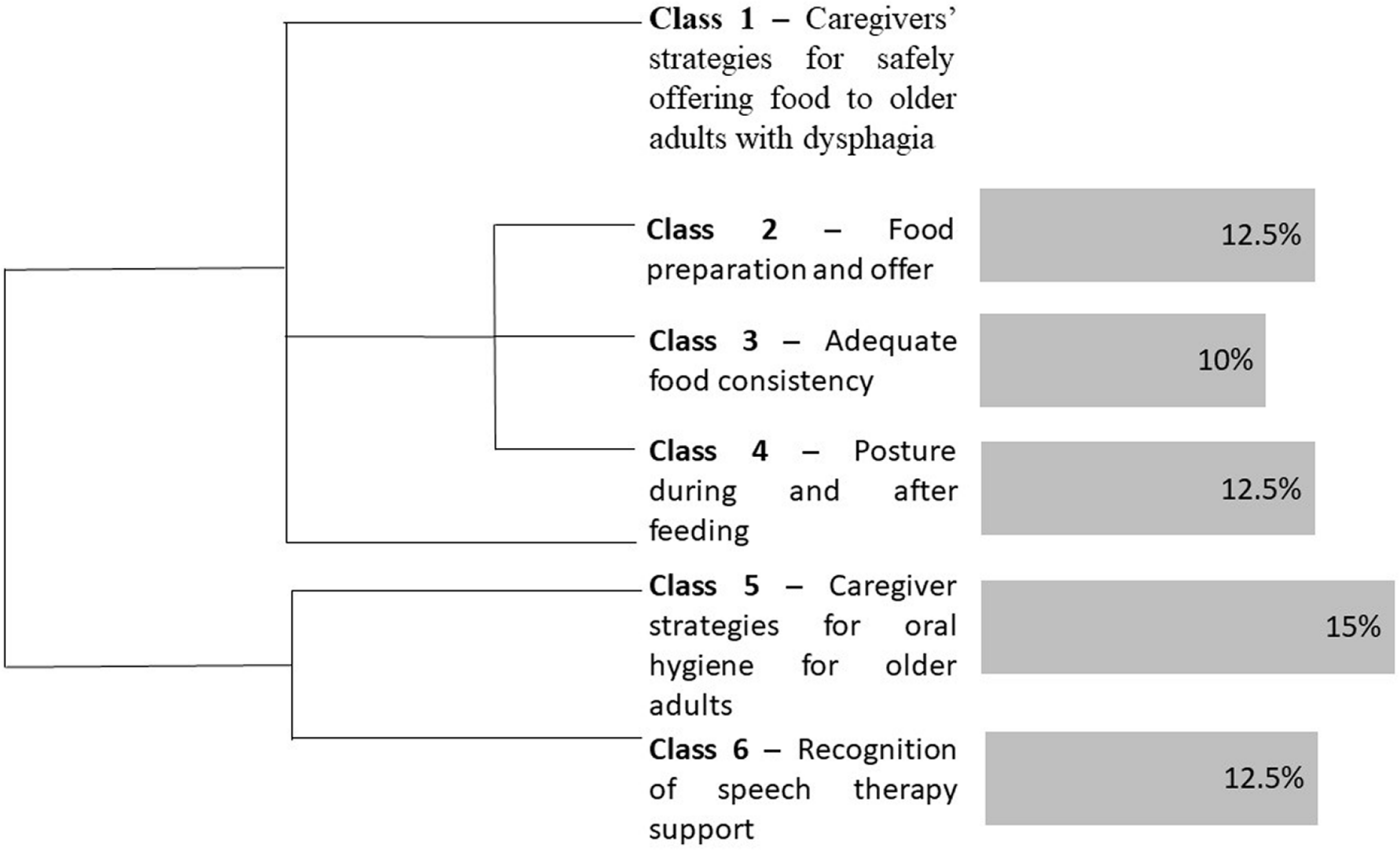
Dendrogram in the Descending Hierarchical Classification of text segments related to the categories that emerged from content analysis prepared by the authors. Salvador, BA, Brazil, 2023.

To understand the strategies adopted by caregivers to offer food to older adults with OPD, the text *corpus* was composed of 45 text segments, emerging 1,672 occurrences (verbs, adjectives, adverbs, words), as 50.35% were distinct and 12.85% were repeated, with 142 having a single occurrence. Interpretative content analysis is presented in the form of categories: 1. “Caregivers’ strategies for safely offering food to older adults with dysphagia”, with the subcategories preparing and offering food (12.5%), adequate food consistency (10%), posture during and after eating (12.5%), obtaining a total of 22 responses; 2. “Caregiver strategies for oral hygiene in older adults” (15%), with seven responses; 3. “Recognition of continuity of speech therapy after dehospitalization” (12.5%), with five responses (Figure 1).

**Figure 2:**
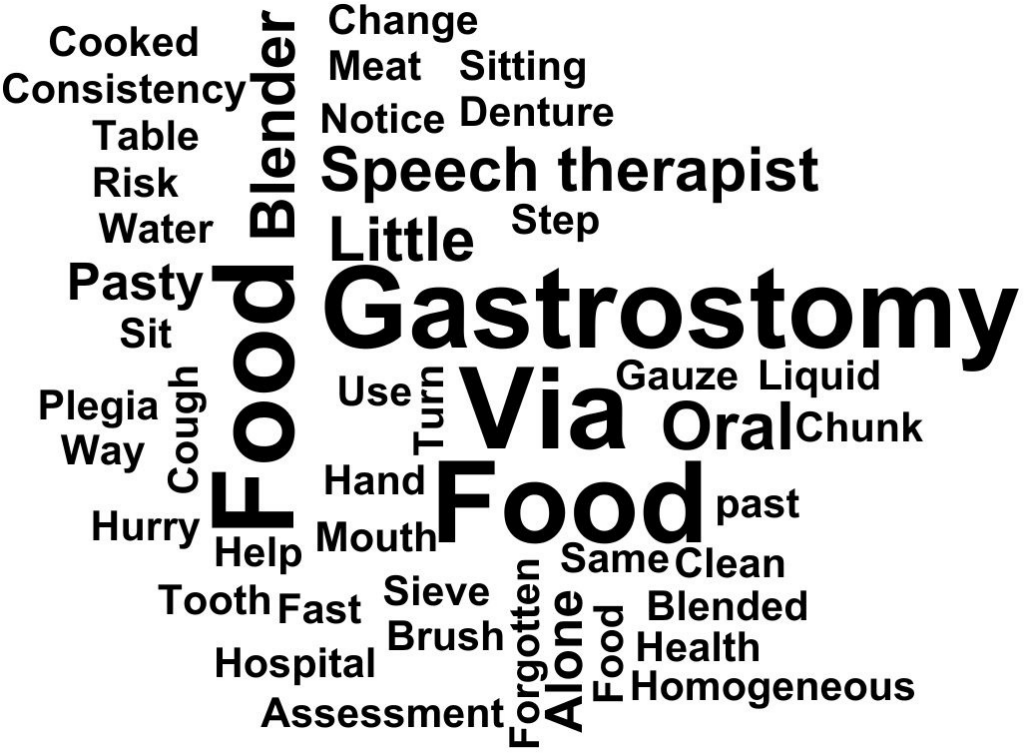
Word cloud about the most mentioned statements by caregivers who experienced offering food to older adults with oropharyngeal dysphagia and its repercussions at home. Salvador, BA, Brazil, 2023.

In order to highlight the words and expressions that appeared most frequently in interviewed caregivers’ speeches, we chose to use the word cloud resource to portray them (Figure 2). It was possible to determine the words with the highest occurrence in the speeches: via (f = 45); feeding (f = 44); gastrostomy (f = 38); food (f = 32); oral (f = 24).

**Figure 3:**
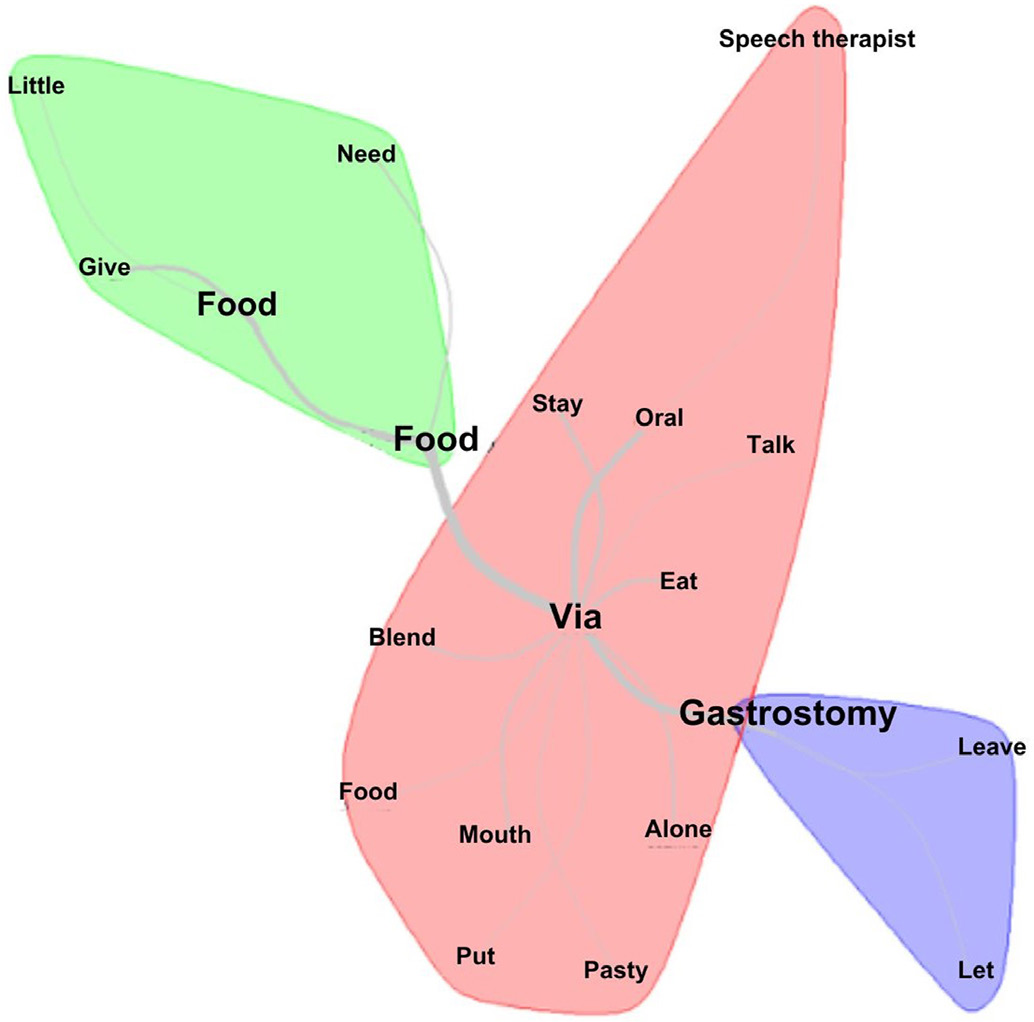
Similarity analysis with the connection and indications of connection between the words related to the statements most mentioned by caregivers who experienced offering food to older adults with oropharyngeal dysphagia. Salvador, BA, Brazil, 2023.

Using similitude analysis based on graph theory, it was possible to deepen the understanding of the relationships between words and their occurrences, in order to identify the structure of the lexical content with connections (links) between them (Figure 3). In this analysis, words with recurrence of at least seven times were included. It was observed that the words that stood out the most were via, gastrostomy, feeding, oral, food, give. The term “via” assumed the central position with the greatest number of connections, which made it possible to generate two groups of equivalent branches, which can be observed by the configuration of the thick lines that express the words with greater recurrence.

Therefore, it can be inferred that caregivers’ statements present references regarding the strategies adopted to safely offer food to older adults with dysphagia, identifying that transition of care was effective, because, although caregivers mentioned that they did not remember the instructions offered throughout the hospitalization, it was noted that, upon returning home, they were able to clearly point out the importance of preparing the food as well as offering it slowly and patiently, as the following text segments reveal:

*I don’t know if I remember the instructions, but I remember that the food should be cooked more, as it helps when swallowing, and always offered with patience and calm.* (Bromeliad – oral feeding route)

*They advised me on the use and handling of the gastrostomy, never to offer the diet quickly so as not to fill the stomach and cause discomfort.* (...) *I already know how to make food; everything is well blended and my hands are always clean. I always give water with a syringe.* (Eleven o’clock– gastrostomy feeding route)

*At the hospital, I was told how to prepare the food; I understood all the instructions and was able to do all the steps of the process at home. After he returned home, his diet needed to change, everything started to be more cooked and blended*. (*Manacá-da-serra* – oral feeding route)

When interpreting the data, based on similarity analysis, it is observed that the thinner lines are the word segments with less repetition, but for the word “speech therapist”, it has a frequency of eight repetitions in participants’ speeches. However, caregivers recognized the need for an assessment by a speech therapist to intervene in dysphagia implications, which was reported as choking or coughing when an attempt was made to return to oral feeding at home. However, they did not know how to access the health network or how to pay for this professional’s care, supporting the previous analysis:

*I understand that if you start choking straight away, it is a sign that the food is going to the wrong hole* (path). *So, I asked for a speech therapist to assess, after that, we were clear about what I could do to make him eat and how to help him speak*. (*Ipê* – oral feeding route)

*Sometimes she asks for water, I give her just a little, and she chokes straight away, so she’s not feeling well yet* (dysphagia). *She doesn’t like seeing food all beaten up, she has asked to eat it orally. I cannot give food through my mouth until it has been evaluated by a professional* (speech therapist), *because there is a risk of the food going the wrong way. The food tests will be done by a speech therapist. Can I do this?* (*Caliandra* – gastrostomy feeding route)

*I know that he should only be fed through gastrostomy, but I have noticed that he swallows normally. So, I started giving him some of the blended food. I do this so he doesn’t lose his sense of taste. Every time I give him some of this soft food, I notice that sometimes he coughs. I’m going to see with my family how we can pay for a speech therapist to assess it.* (*Alamanda* – gastrostomy feeding route)

**Figure 4:**
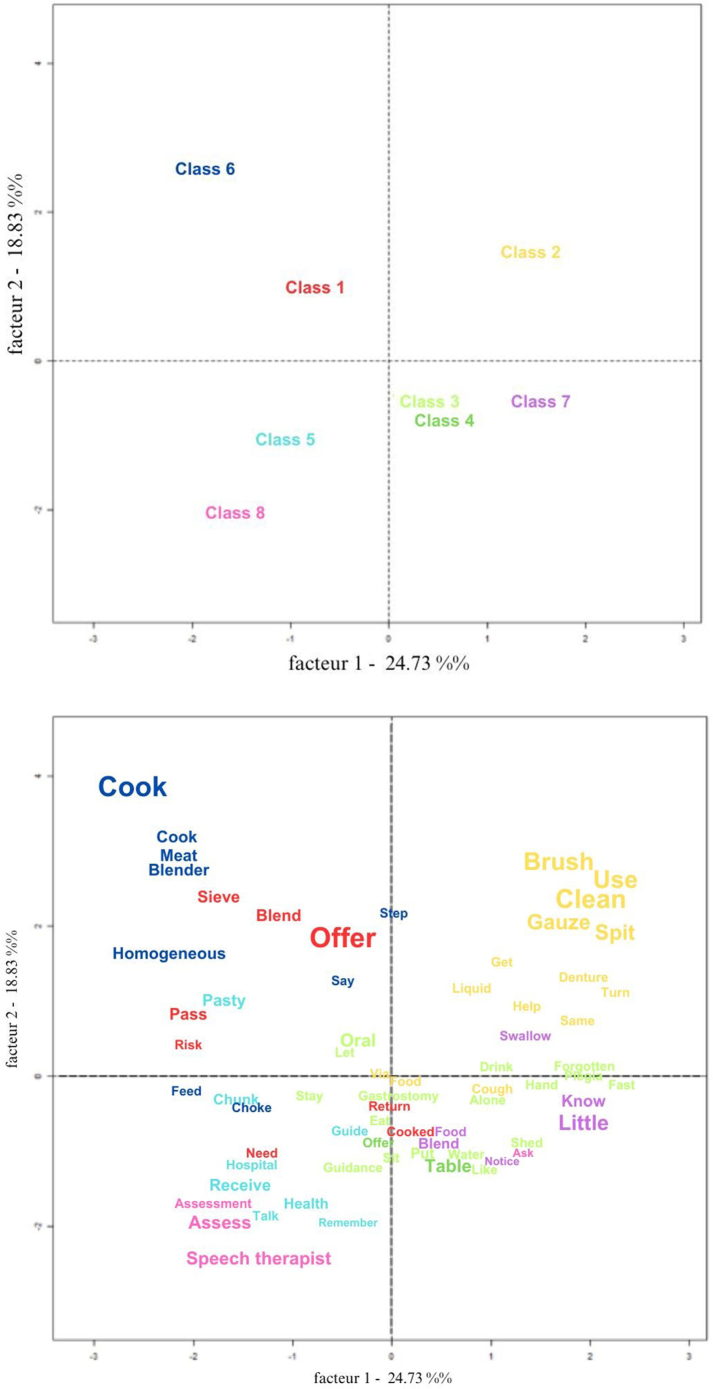
Correspondence Factor Analysis of text segments related to caregivers’ statements regarding care for older adults with oropharyngeal dysphagia. Salvador, BA, Brazil, 2023.

In CFA analysis, some words appear more centralized, while others project to the periphery. It is noteworthy that the upper quadrants (right and left) in orange and blue are the most isolated in the Cartesian plane, represented by classes 2 and 6, identified in Figure 4.

In interpretative analysis, it was found that caregivers perceived the ideal consistency of the food and used instruments (blender and sieve) to obtain a homogeneous pasty consistency, in order to avoid choking and/or coughing, according to text segments:

*She feeds orally, but they are foods with a homogeneous pasty consistency. She never choked on liquid consistencies. The change was in the meat, they said to cook it more, or put it in the blender so she could eat it without difficulty. Everything is blended to obtain a homogeneous pasty consistency.* (May Flower - oral feeding route)

(...) *all of his food is well blended! I steam everything, so the food doesn’t lose its nutrients. I always cook brown rice; I cook the beans together with the muscle meat. I let the meat cook together to leave its nutrients. The juice, always natural from the fruit, I blend in a blender and pass through a sieve.* (Eleven o’clock - gastrostomy feeding route)

*I make food every day so he can eat healthy things* (...) *the food should be better cooked and I try to offer liquids. He doesn’t like drinking water, and he complains a lot!* (Bromeliad - oral feeding route)

It is also possible to state that class 2 represented in orange shows that caregivers’ strategies for oral hygiene for older adults are carried out with the help of a brush and gauze. A good understanding was observed regarding oral care, such as cleaning the mouth, tongue and removing dentures in cases of use. Participants also revealed that older adults should be careful not to ingest the contents in their mouths, advising them to expectorate. The statements below reveal the strategies:

*They advised me at the hospital and I’m doing it. He only eats via gastrostomy. As he is no longer using his dentures, I put the glove on my hand, take the gauze, apply a little mouthwash and squeeze the gauze a lot to make it moist, then I put it around his mouth, asking him to put his tongue in out to clean.* (*Alamanda* – gastrostomy feeding route)

*I wasn’t used to cleaning his dentures, now I ask him to open his mouth, sometimes there’s food, I clean it! I use a brush or gauze to clean. We took off his dentures because they were very loose and we were afraid he would swallow them.* (*Manacá-da-serra* – oral feeding route)

*The guidelines were also about maintaining mouth hygiene,* (...) *he can clean his teeth on his own. He brushes and doesn’t drink, he spits everything out, because if he drinks the liquid, he coughs.* (Rosewood – gastrostomy feeding route)

In classes 3 and 4, represented in dark and light green colors, we can infer that caregivers understand the importance of proper posture for eating, placing older adults in a sitting position, with the help of pillows, or when an older adult has the possibility of eating at the table with family members. They also realized the importance of encouraging them to feed themselves. The statements below illustrate the meaning of posture during and after eating.

(...) *now he eats alone with his left hand, because the other arm is forgotten* (plegia), *he gets a little dirty, but even so, I let him eat alone. I sit him at the table to eat with us, he eats, drinks alone, and is happy!* (*Ipê* - oral feeding route)

*We placed him sitting on the bed with the support of a pillow to feed him through gastrostomy. When it’s over, we don’t put him to bed right away so the food doesn’t return.* (*Alamanda* – gastrostomy feeding route)

*When she eats alone, the food spills more, we have the habit of giving her food, but we also let her put a spoon in her mouth and she eats alone. I sit her at the table, she picks up things with one hand, the other hand is forgotten* (plegia). (May Flower - oral feeding route)

## DISCUSSION

It is observed that initial inducing stimulus focused on “offering of food by caregivers to people with OPD” made it possible for the analysis to transcend this specific issue of the study. This suggests that participants’ responses were more comprehensive and involved aspects beyond the scope of caregiver strategies after dehospitalization. Dealing with OPD requires a multidisciplinary approach and a partnership between healthcare professionals, caregivers and older adults. Careful planning and maintenance of health education are fundamental components to ensure an effective transition of care after hospital discharge and, thus, provide caregivers with safe management of dysphagia at home.

The study showed a predominance of females, who declared themselves black, playing the role of caregivers. Gender and race issues are intrinsically related to historical, political, social, economic and cultural issues and reaffirm the unequal distribution of care responsibility^(15)^. The importance of addressing these issues such as gender and racial equity in access to care and support for caregivers stands out, as they are not burdened by unpaid care^(5)^.

The factorial representation provided by IRaMuTeQ confirmed the interconnection of the classes. This convergence between qualitative analysis and statistical approach reinforces the validity and robustness of the results obtained. Based on data analysis, the observation that some caregivers do not remember the instructions provided during hospital discharge is significant. This raises important questions about understanding and retaining care practices during the hospitalization process. The immediate memory of guidelines may be inconsistent, but the tacit and practical knowledge of general care regarding older adults’ nutrition is linked to this knowledge, supported by the elaboration of strategies developed during patient hand-off from hospital to home, giving greater security to this supply, ensuring control of rhythm, volume, posture and food consistency^(7,8,10,11)^.

Caregivers understood the importance of preparing food with a longer cooking time, in order to guarantee homogeneity and adequate supply using utensils to promote this texture. Supporting the findings, studies reveal that caregivers recognize the need to use tools, such as blenders and sieves, to obtain a pasty and homogeneous texture, minimizing the risk of choking or coughing during feeding^(5,16)^. This demonstrates genuine concern for older adults’ safety and comfort while eating^(9)^.

In CFA analysis, the terms “liquid” and “cough” are arranged in class 2 (Figure 4), approaching the center. In this directive, the semantic contents in question can be related to precautions for drinking fluids. It is inferred that refusal to drink liquids by some older adults can lead to dehydration problems, impacting their health in general. Caregivers who understand the importance of safe daily water intake for older adults are alert to possible signs of an inadequate supply^(17,18)^. In this study, an older adult presented restrictions on oral food consistency and refused to drink liquids. Caregivers had reported that, before hospitalization, there was already this refusal even after trying to taste the water. Not drinking fluids leads to skin dehydration and can affect the brain functioning, leading to confusion, difficulty concentrating and cognitive impairment, as well as causing muscle weakness and dizziness, increasing the risk of falls^(17,18,19,20)^.

It is important to consider the safety of offering fluids to older adults suffering from dysphagia, as inadequate intake can cause health problems. Each strategy adopted during the offer varies depending on individual needs. Therefore, it is essential that caregivers are guided and trained by healthcare professionals even during hospitalization. It is necessary to emphasize, at this point, the risks that liquid consistency can pose, and, if necessary, liquids can be thickened to a specific consistency, such as nectar, honey or pudding, which makes them safer for swallowing^(17,19)^. Although there is national^(6,21)^ and international^(20)^ production aimed at caregivers in the presence of dysphagia, it is important to adapt guidelines on nutrition to individual reality. Therefore, interventions must consider not only the pathological condition, but also the particularities of dysphagia and other specific health needs and preferences of people and their caregiver, if applicable, ensuring not only food security, but also quality of life and satisfaction during meals.

The different text processing alternatives offered by the software made it possible to identify caregivers’ attention to oral hygiene, using a brush, gauze and mouthwash. Some participants used dental prostheses, and their caregivers were knowledgeable about this care, sharing responsibility with older adults and ensuring individual needs were met to avoid problems such as cavities, gum disease and infections^(22,23)^.

Caregivers were concerned about proper posture before and after feeding. The sitting posture facilitates chewing and swallowing, preventing choking and improving the effectiveness of digestion. Furthermore, it reduces the risk of acid reflux and gastric discomfort after meals^(18,22,23)^. International studies indicate that placing older adults sitting in bed with the help of pillows or at the table next to family members is a careful and attentive approach that ensures the appropriate posture for receiving food^(24,25)^. Guidance regarding posture is relevant, as, in the case of bedridden people, it is necessary to guide chest elevation, especially in situations of dysphagia as well as careful oral hygiene. As for sitting at the table, it is necessary to pay attention to trunk balance. Therefore, there is a need for care for a safe transition throughout hospitalization.

In class 4, it was observed that the positive perception of encouraging independence and autonomy by caregivers is essential to promote a sensitive approach focused on older adults’ individual needs and capabilities. Allowing them to actively participate in eating can help maintain their motor skills, coordination, and sense of control over daily activities^(16)^. This not only promotes autonomy and independence, but also cognitive and emotional benefits. This stimulus preserves dignity and quality of life while promoting a healthy relationship between caregiver and person being cared for^(1,16)^.

In class 8, it is noted that the term “speech therapy” appears closer to the center, making it possible to infer, given the speeches, that there is a worrying gap in health services, especially related to the lack of speech therapy professionals. The lack of access to speech therapists has significant consequences for continuity of care^(2,8)^. The anguish and insecurity that surrounds people in this situation are understandable, since the inadequate introduction of food orally can pose a serious risk of undesirable events^(2,16)^.

It is known that the allocation of adequate human resources and the hiring of qualified professionals are essential to guarantee an effective approach to assess, diagnose and treat issues related to eating and swallowing^(2)^. Strengthening health teams with the presence of speech therapy professionals has a significant impact on reducing the risks faced by older adults during oral feeding introduction, contributing to reducing rehospitalization rates and better management of dysphagia^(2,26)^.

Specialist guidance reduces the risks associated with this and other related problems, as these professionals have specific knowledge to assess, diagnose and treat issues related to eating and swallowing^(2)^. Considering the difficulty in this monitoring, one option would be remote care, which would expand access and support to caregivers even in rural areas or regions with limited resources^(27)^. This type of care can clarify caregivers’ doubts, serving as a home support strategy. Although distance health services, such as online consultations or telecare, offer significant benefits, such as greater accessibility to the health service, convenience and reduction of geographic barriers, they cannot replace the professional’s in-person assessment, which is more complete and detailed^(27)^. However, it can contribute to a broader system of care for older adults.

Therefore, support for caregivers is essential to mitigate the challenges faced and ensure their well-being and that of the people they care for^(1)^. Considering this observation, it is also necessary to develop support and training strategies that reinforce caregivers’ tacit knowledge, recognizing and strengthening their daily practices. Hence, creating educational materials, such as booklets, manuals and videos, can help, taking into account the cultural and regional context. It is also necessary to develop public policies, programs and services that meet the specific needs of these people.

As a limitation, the identification of the sociodemographic and cultural homogeneity of the studied population is highlighted, consisting of older adults with neuromusculoskeletal disorders and their caregivers, assisted by the Brazilian Health System, linked to a specific region of the country. However, the study responds to a lack of qualitative research in the area of health and speech therapy on the topic, increasing its visibility.

## CONCLUSION

The strategies used by caregivers to offer food to older adults with OPD were related to how to prepare and offer food, especially when it is through gastrostomy, pre- and post-feeding oral hygiene, adequate posture during and after feeding, in addition to encouraging them to eat alone, the ideal consistency of the food and the importance of maintaining speech therapy, in order to avoid coughing and/or choking. Caregivers’ tacit knowledge of caregivers supported the development of strategies. It is clear that the health service guaranteed a smooth transition and continuity of care, in addition to recognizing that caregivers play a fundamental role in the process of maintenance and recovery of older adults.

Given the insufficient number of speech therapists in healthcare teams, which negatively impacts the quality of care provided to hospitalized older adults and the guidance provided to caregivers, it is suggested to create new services, with the presence of speech therapy, at different levels of healthcare that assist or accompany older adults with OPD after hospital discharge in their own homes.

The proposal to guide and train caregivers during older adults’ hospitalization process, followed by telemonitoring carried out by healthcare professionals after hospital discharge, is a comprehensive approach that aims to guarantee continuity of care remotely. Therefore, preparing educational materials according to the specific profile, caregivers’ experience and older adults’ health needs can promote safety in provision of care at home by this caregiver. These materials can cover information about dysphagia, safe feeding techniques, signs of complications, among other relevant aspects.

New research is suggested with the aim of reinforcing telemonitoring as a tool capable of playing a significant role, providing continuous training and guidance to caregivers, allowing learning to be accessible and flexible. By integrating telemonitoring in a way that is sensitive to the specific needs and contexts of caregivers and older people, it is possible to improve efficiency of care, promote communication between among healthcare team members, caregivers and older people, and offer more comprehensive quality and personalized support.
